# When Science Cannot Guide us: A Call to Action for Applied Behavior Analysts

**DOI:** 10.1007/s40614-023-00377-y

**Published:** 2023-06-06

**Authors:** Dorothea C. Lerman

**Affiliations:** University of Houston-Clear Lake, Houston, TX USA

**Keywords:** ABAI position statement, Contingent electric skin shock, Ethical issues, Severe problem behavior, Treatment-resistant behavior

## Abstract

Evidence presented in the ABAI Task Force Report on Contingent Electric Skin Shock (CESS) revealed serious ethical, clinical, and practical problems with the contemporary use of CESS. As a member of the task force, I ultimately concluded that our recommended position statement (“Position A”) was a misguided attempt to uphold the field’s commitment to client choice. Furthermore, information gathered by the task force supports an urgent call to find solutions to two additional troubling issues: a severe shortage of treatment services for severe problem behavior and the near-absence of research on treatment-resistant behavior. In this commentary, I discuss reasons Position A was not a tenable stance and why we must do better to help our most vulnerable clients.

The controversy surrounding a single institution’s use of contingent electric skin shock (CESS) to control the behavior of clients was a call to action for behavior analysts. However, passage of a position statement that opposes rather than preserves the use of CESS is just the first step. Evidence in the task force report not only supports a position opposing the use of CESS but indicates that our work is far from finished. This call to action also must address easily overlooked facts contained in the task force report regarding access to contemporary, state-of-the-art treatment for severe destructive behavior and the status of research on treatment-resistant behavior. In this commentary, I will discuss the reasons that the now-defeated position statement recommended by the task force (i.e., “Position A”) was not a tenable stance and why applied behavior analysts must do additional work to best serve our most vulnerable clients.

The central tenet of Position A was that an individual should have the right to choose the most effective treatment procedure available (Van Houten et al., 1988). However, this venerable and important right must be applied in an ethical manner. This right should not supersede our ethical mandate to protect all clients from harm; to treat our clients with compassion, dignity, and respect; and to provide evidence-based services that are conceptually consistent with behavioral principles. With this in mind, Position A embedded the right to choose CESS within a set of highly restrictive conditions, including a requirement to continually ascertain the assent of the person receiving CESS.

In the end, a significant majority of ABAI members who cast votes, including myself, chose to support a position statement that opposed the use of CESS under any condition. Although a multitude of factors likely determined members’ decision, my shift away from Position A occurred upon further reflection of the evidence provided by both the science and practice of CESS. My decision was further solidified as I thoroughly considered the following questions: What types of evidence are necessary and sufficient to support the clinical use of CESS? What constitutes “effective treatment?” And under what conditions should we limit an individual’s access to CESS? This reflection led me to conclude that Position A was a misguided attempt to uphold the field’s commitment to client choice. In doing so, it explicitly endorsed a default technology with serious ethical, clinical, and practical challenges.

## Can Science on CESS Guide us?

An in-depth review of the existing evidence on CESS led the task force to conclude, “Research and practice in applied behavior analysis is largely silent on questions about best practices for implementing CESS, ensuring the safety of clients, and fading CESS out of a treatment plan or removing it altogether” (p. 9>). In fact, our knowledge about CESS is particularly scant. It is not surprising that the limited research on CESS has shown that contingent shock of sufficient intensity can be highly effective in suppressing behavior to low levels. However, the task force report also noted that few studies have evaluated the long-term outcomes or side effects of CESS and that no studies have evaluated strategies for transferring control from CESS to other, less intrusive procedures. Most concerning, a retrospective analysis of clinical data collected across 20 years for 173 clients at the only facility to routinely use CESS found that it could be faded completely for just 27% of clients after more than 5 years of this treatment (Yadollahikhales et al., 2021). Most of these clients have continued to receive CESS indefinitely because the targeted behaviors increase when clinicians attempt to reduce or remove CESS from their treatment plans. This poor long-term prognosis is what led some scholars and professional associations like ABAI to question the effectiveness of CESS (e.g., Zarcone et al., 2020; see position statements of ABAI, Association of Professional Behavior Analysts, KansABA Executive Council).

It could be argued that the evidence for other types of behavior-analytic treatments is similarly incomplete and that extinction effects demonstrate the “temporary” nature of reinforcement-based treatment. I would argue that all stimuli should not be considered equivalent. CESS, in particular, must be held to a higher standard because it involves the deliberate application of physical pain, typically across many years and possibly for the client’s lifetime; it is being offered to a highly vulnerable population; and it is a controversial treatment that is unlawful in many states and countries.^1^ Clients receiving this treatment may be especially vulnerable to the inappropriate or unnecessary use of CESS because it likely reduces the urgency to invest time and resources in the pursuit of less intrusive but effective procedures. Few would argue that a lifetime of CESS is comparable to a lifetime of reinforcement.

Most important, contemporary CESS practices, as outlined in the task force report and discussed further in the next section, bear little resemblance to those evaluated in the controlled study of CESS. This includes the intensity, duration, immediacy, and consistency of the stimulus, and the targeted topographies of behavior. In fact, our science has provided little guidance on these contemporary CESS practices and cannot support their use. These practices violate our ethical obligation to provide evidence-based services that are conceptually consistent with behavioral principles.

The dearth of research on CESS, however, should not lead us to call for more research on CESS. The discipline has long shifted its focus away from default technologies and towards function-based intervention. A call for more research on CESS is not only an ill-advised distraction from the scholarly pursuit of more compassionate solutions for treatment-resistant behavior, but it is unlikely to be heeded after more than 20 years of dormancy.

## Other Evidence to Guide us: An Organization as Case Study

Beyond the scholarly literature, what other evidence might help to guide us? The task force report noted that one facility, the Judge Rotenberg Center (JRC), has incorporated CESS as part of their clinical work for more than 20 years. JRC thus offers a wealth of descriptive data about the use of CESS, including both the outcomes for clients and for the provider itself. That is, the experiences of JRC could function as a type of case study that sheds some light on the consequences of adopting this technology and pursuing caregivers’ right to choose it. In the summer of 2022, staff at the JRC provided task force members with nearly unfettered access to client records with guardian consent, including court-approved treatment plans, and to other relevant documents (policies, procedures, court records). They arranged for task force members to speak with clinicians, clients, parents, and other JRC-affiliated professionals during a 2-day visit and to experience the physical stimulation used on clients. Information gathered as part of these activities was included in the task force report.

This information revealed a number of current practices at the JRC that concerned task force members. These troubling practices have continued despite decades of scrutiny and legal challenges to those practices (see Nisbet, 2021, for a densely referenced historical record). The restrictions listed in Position A were largely a reaction to concerns about these questionable practices. At least 7 of the 12 restrictions were explicitly intended to change practices at the JRC.^2^

Among the most concerning, JRC staff apply CESS to a wide range of behaviors deemed “harmful” but not severe or life-threatening, such as noncompliance, yelling, removing hands from a holster, and defecating outside of the toilet. In court-approved treatment plans, these relatively minor behaviors are described as harmful because they “interfere with educational or social development,” or because they are presumed to be precursors to or in the same response class as more severe behavior (e.g., self-injury, aggression). The latter rationale for targeting these minor behaviors with CESS is particularly problematic because (1) JRC staff reported conducting very few functional analyses; (2) none of the reviewed records or treatment plans included empirically based assessments of precursors or response classes (see Heath and Smith, 2019, for a review of these assessments); and (3) little clinical evidence exists to support this approach to treatment.^3^ Further complicating the ethics of such an approach, it seems unlikely that some of these minor behaviors precede the more severe topographies 100% of the time. The result is that clients are shocked anytime that they engage in these behaviors (or, in the case of noncompliance, lack of behavior) for many years and potentially for the remainder of their lives.

A second primary concern is that the contingent application of shock at the JRC can be delayed for as much as 2 min (and up to 30 min for some clients) and occur intermittently for safety and practical reasons. Although these practices may be well-intentioned, results of basic and applied research on delayed and intermittent punishment overwhelmingly indicate that this practice likely reduces the efficacy of CESS. Not only could this ultimately expose clients to more shock than is necessary, it could lead to the inadvertent punishment of appropriate behavior that occurs during the resulting delay.^4^

Third, as noted previously, the majority of clients have continued to receive CESS indefinitely due to difficulties fading the device. This is particularly disconcerting because the task force’s review of client records indicated that some clients have continued to receive many shocks. Finally, JRC staff reported that they had to increase the intensity of shock delivered by their devices (to 15 mA and 41 mA) because the lower level evaluated in the most recent studies on CESS (3.5 mA) was ineffective for the majority of their clients.^5^

In addition to these consequences for clients who receive CESS, information available to the task force revealed that JRC has had to invest a substantial amount of time and resources to ensure that their clients have legal access to CESS. These legal battles with state regulators are well-documented (Nisbet, 2021). Like the JRC, providers of CESS likely will encounter new or more restrictive laws in their states or countries as lawmakers react to complaints from those who deem CESS a socially invalid approach to treatment. In short, even providers who feel strongly that clients should have the right to choose CESS will find it neither practical nor legally possible to offer it.

## Call to Action #1

During the course of its information-gathering, the task force noted two troubling issues that might help explain the continued use of CESS despite various clinical, ethical, and practical challenges. First, interviews with caregivers during the JRC visit and with practitioners from other treatment programs revealed a serious shortage of high-quality behavior-analytic services for those with the most severe behavior disorders. Interviews with caregivers during the JRC visit indicated that these families did not have access to state-of-the-art behavioral treatments prior to their enrollment in JRC. Nationally recognized providers of specialized treatments for severe behavior disorders reported having a limited number of slots and lengthy waiting lists. Although it is difficult to estimate the level of unmet need, it is likely that a majority of those with severe behavior disorders are unable to access these services. As a result, many individuals are likely exposed to highly restrictive procedures, including chemical and physical restraint. The right to choose the most effective treatment presumes that the individual truly has a choice. Thus, restrictions on clients’ and caregivers’ ability to choose from all potentially effective alternatives hinder the ethical application of this right.

Our field should formally recognize this shortage as a serious health care crisis. We need to evaluate the causes and consequences of this shortage. Armed with this information, we should respond with concerted efforts to resolve this unmet need. This likely will require coordinated action among university training programs, nationally recognized treatments centers, and community ABA providers. For example, the task force learned that many ABA providers do not serve those with severe behavior disorders because their clinicians lack the necessary expertise. University programs should work to resolve this problem by broadening the scope of their curriculum, recruiting faculty with the relevant expertise, and providing resources for current faculty to obtain specialized training. Faculty, students, and practitioners might complete short-term internships and fellowships at specialized training and clinical programs. These programs might then offer additional support to trainees as they expand their services to treat clients with severe behavior disorders. Beyond a lack of qualified providers, other causes of this health care shortage will need to be identified and addressed. Advocating for changes to public policy and identifying new funding sources might be crucial for improving access to care.

To illustrate, two prominent scholars in behavior analysis, Greg Hanley and Wayne Fisher, are working to address the shortage of services for severe behavior disorders. Greg Hanley and his consultants provide structured trainings in assessment and intervention to teams of practicing behavior analysts across the globe.^6^ Wayne Fisher has developed a toolkit and accompanying webinar to help guide behavior analysts through the financial and clinical aspects of establishing specialized treatment services.^7^

## Call to Action #2

A second troubling finding identified by the task force is the scarcity of behavior-analytic research on treatment-resistant behavior. Recent large-*N* studies on the effectiveness of behavioral treatments have reported impressive outcomes for the majority of those participating (e.g., Greer et al., 2016; Jessel et al., 2018; Phillips et al., 2017; Rooker et al., 2013). However, current behavioral technologies are not effective in every single case. Further research is needed for the small percentage of clients who do not respond adequately to gold-standard treatments. A few recent examples of this work illustrate ways to approach this problem. For example, Louis Hagopian has established a line of research to understand the mechanisms underlying automatically reinforced problem behavior and to predict responsiveness to different behavioral treatments (Hagopian et al., 2018). Tim Vollmer and his students are developing a model for ruling out potential medical complications that underly treatment-resistant behavior (T. Vollmer, personal communication, August 15, 2022).

However, the existence of treatment-resistant problem behavior is not an appropriate justification for using CESS with vulnerable clients and it should not lead us to endorse it as an acceptable last resort. Evidence contained in the task force report indicates that by doing so, we risk exposing our clients to the inappropriate use of a highly painful procedure, possibly for the rest of their lives. Although some may argue that nonbehavioral alternatives for those with treatment-resistant behavior (e.g., sedation, restraint) are no more ethical than CESS, this fact also should not lead us to advocate for the use of CESS. A position statement opposing use of a procedure with clear clinical, ethical, and practical difficulties does not constitute approval of the alternatives. The existence of treatment-resistant behavior should urge us to find compassionate solutions to the problem.

## We Must Do Better

Together, the shortage of treatment services for severe problem behavior and the problem posed by treatment-resistant behavior should lead us to vigorous action. They should encourage us to invest as much passion, time, and energy on resolving these problems, and to express as much outrage about the existence of these problems as we did in opposition to CESS itself. These problems should not deter us from following our ethical mandates to do no harm; to treat our clients with compassion, dignity, and respect; and to provide evidence-based services that are conceptually consistent with behavioral principles. They should not lead us to endorse problematic default technologies. We simply must do better. The lives and welfare of our clients and the future of our field depend on it.

## Data Availability

Data sharing not applicable to this article because no datasets were generated or analyzed during the current study.

## Footnotes

1 CESS is even illegal in the home state of the only facility in the country using CESS; a consent decree permits them to circumvent this law by gaining approval from a state court on a case-by-case basis.

2 I ultimately concluded that these restrictions came with their own set of insurmountable hurdles, a sentiment that also was expressed by ABAI members during the comment period.

3 In fact, research supports the opposite approach; reinforcing precursors to severe problem behavior during functional analyses and pretreatment baselines has been shown to decrease the likelihood of the more severe forms of behavior (Dracobly & Smith, 2012; Fritz et al., 2013).

4 This problem is not mere speculation. Some JRC treatment plans reviewed by task force members specified that staff should deliver shock even if the client complies with a directive during the delay.

5 The majority of studies on CESS conducted since 1980 evaluated the Self-Injurious Behavior Inhibiting System (SIBIS), a device that automatically delivered a relatively brief, mild stimulus contingent on severe self-injury (e.g., Linscheid et al., 1990).

6 https://ftfbc.com/

7 https://assets.foleon.com/eu-west-2/uploads-7e3kk3/25315/severe-behavior-program-toolkit.90e222c3f2d1.pdf
